# Editorial of the Special Issue: Antisense Therapies

**DOI:** 10.3390/biomedicines6040095

**Published:** 2018-09-27

**Authors:** Linda J. Popplewell

**Affiliations:** Centres of Gene and Cell Therapies and Biomedical Sciences, School of Biological Sciences, Royal Holloway University of London, Egham, Surrey TW20 0EX, UK; Linda.Popplewell@rhul.ac.uk

In this special issue of Biomedicines focusing on Antisense Therapies, we include insightful reviews on the state-of-the-art in relation to a number of specific diseases (in particular, Duchenne muscular dystrophy (DMD) [1,2,3], amyotrophic lateral sclerosis (ALS) [4], and fibrosis [5]), and also on issues that need addressing and the steps being made to overcome them.

Antisense oligonucleotides (AOs) were first developed in 1977, and with advances in the technology have been utilised to inhibit 5’ cap formation, sterically block translation, alter pre-mRNA splicing, or activate RNase H. Because of their wide-ranging mechanisms of action, AOs have diverse therapeutic applicability. As such, several AOs have been approved by the FDA for clinical use against a number of disease indications. These include (i) Formivirsen, which targets the immediate early 2 gene for the treatment of cytomegalovirus retinitis (CMV) in immunocompromised patients, including those with acquired immunodeficiency disease (AIDS) (approved 1998); (iii) Macugen, which binds to extracellular vascular endothelial growth factor to prevent age-related macular degeneration, one of the primary causes of blindness (approved 2004); (ii) Mipomersen, which targets apolipoprotein B-100 for the treatment of homozygous familial hypercholesterolemia (approved 2013); (iv) Eteplirsen, which restores the transcript reading frame through inducement of exon skipping in certain Duchenne muscular dystrophy (DMD) patients (approved 2016); (v) Defitelio, whose mechanism of action is complicated but involves interaction with FHF2, which is used for the treatment of severe hepatic veno-occlusive disease (sVOD) (approved 2016); and (vi) Spinraza which induces the inclusion of exon 7 in the SMN1 and SMN2 mRNA by targeting and blocking an intron 7 internal splice site and is used in infants with types 1, 2, and 3 spinal muscular atrophy (SMA) (approved 2016).

Despite these milestone approvals, there still exist a number of hurdles that need tackling to allow AOs to achieve their full potential. These include enhancing the delivery of AOs and improving the biological efficacy of action, as well as reducing the toxicological impact and financial cost of AO therapies. Advances are being made to address poor delivery and efficacy (and by implication the potential toxicology and financial burden) through the use of new chemistries and conjugated AOs. Reviews covering a newer chemistry of AO, tri-cycloDNA [1], and AOs conjugated to cell-penetrating peptides [3] are therefore included in this special issue.

AOs are increasingly being used to manipulate pre-mRNA splicing as a means to treat specific disease mutations. Where a disease is caused by an array of mutations, the development of an AO that could treat all patients would be impossible. However, steps are being made to enhance patient applicability. Using DMD here as an example, AOs have the potential to restore the transcript reading frame for 69% of patients with deletion mutations through the skipping of single exons. If skipping of multiple exons were possible, then this would increase the patient applicability to 90%. The most recent advances in the field in relation to the development of multi-exon skipping is included in this special issue [2]. As well as addressing the primary genetic defect, AOs can target other genes involved in pathological processes such as the muscular atrophy and muscular fibrosis associated with DMD [5].

On the basis of recent clinical approvals linked with the advances made pre-clinically in improving efficacy and applicability, it is likely that AOs have much still to deliver.
